# Puke or poop? Comparison of regurgitate and faecal samples to infer alpine grasshopper (*Paprides nitidus* Hutton) diet in experimental plant communities

**DOI:** 10.1002/ece3.10444

**Published:** 2023-08-29

**Authors:** Warwick J. Allen, Lauren P. Waller, Barbara I. P. Barratt, Ian A. Dickie

**Affiliations:** ^1^ Bio‐Protection Research Centre, School of Biological Sciences University of Canterbury Christchurch New Zealand; ^2^ Bioprotection Aotearoa Lincoln University Lincoln New Zealand; ^3^ AgResearch, Invermay Research Centre Mosgiel New Zealand; ^4^ Department of Botany University of Otago Dunedin New Zealand; ^5^ Present address: Manaaki Whenua ‐ Landcare Research 76 Gerald Street Lincoln 7608 New Zealand

**Keywords:** eDNA, environmental DNA, frass, plant‐herbivore interactions, restriction fragment length polymorphism, RFLP

## Abstract

Characterising plant‐herbivore interactions is important to understanding the processes that influence community structure and ecosystem functioning. Traditional methods used to identify plant‐herbivore interactions are being superseded by non‐destructive molecular approaches that can infer interactions with greater resolution and accuracy from environmental DNA (e.g. faeces and regurgitate). However, few studies have compared the success of using different sample types and whether they provide similar or contrasting information about species' diet. Here we compared the success of DNA amplification and host plant species identification using restriction fragment length polymorphism (RFLP) applied to faecal and regurgitate samples collected from alpine grasshoppers *Paprides nitidus* Hutton during a grassland community mesocosm experiment. We found that DNA amplification success was 23% and 86% higher for faecal than regurgitate samples from female and male grasshoppers, respectively. In contrast, successful host plant identification using RFLP was 9% higher for regurgitate than faecal samples. The mean number of host plant species identified per sample (1.40) did not differ between sample types or grasshopper sexes. Of the 136 paired faecal‐regurgitate samples, just 41% and 74% produced exactly or partially matching host plant identifications, respectively, indicating that different sample types provided complementary information about herbivore diet. Some plant species were more likely to be identified from faecal samples than expected by chance, and we found that this identification bias skewed towards plant species with higher investment in leaf tissue. We conclude that multiple sample types may be required to fully characterise an invertebrate herbivore species' diet.

## Graphical abstract

We compared the success of DNA amplification and host plant species identification using restriction fragment length polymorphism (RFLP) applied to faecal and regurgitate samples collected from alpine grasshoppers *Paprides nitidus* Hutton during a grassland community mesocosm experiment. We found that DNA amplification success was 23% (female grasshoppers) and 86% (male) higher for faecal than regurgitate samples, whereas successful host plant identification using RFLP was 9% higher for regurgitate than faecal samples, regardless of grasshopper sex. Importantly, faecal and regurgitate samples provided complementary information about herbivore diet, and some plant species were disproportionately represented in faecal samples, indicating that multiple sample types may be required to fully characterise an invertebrate herbivore species' diet.

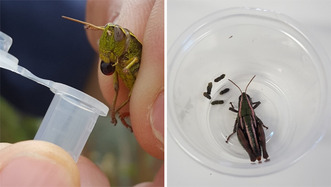

## 1 INTRODUCTION

Plant–herbivore interactions link primary productivity with food webs and play a central role in regulating community structure (Agrawal & Maron, 2022; Brown & Gange, 1992; Kempel et al., 2015), ecosystem functioning (Belovsky & Slade, 2000; Waller et al., 2020) and ecosystem service provision (Schowalter, 2012). To better understand these ecosystem processes and properties, it is important to be able to accurately characterise plant–herbivore interactions, but this can be challenging to achieve since herbivory is a dynamic process that is not always easy to observe. Traditional methods have inferred plant–herbivore interactions through field observation of feeding or co‐occurrence (Novotny et al., 2002; Zhu et al., 2019), morphological identification of plant tissue from gut contents or faecal (frass) samples (Garnick et al., 2018; McInnis et al., 1983), laboratory feeding trials (Barone, 1998) and stable isotope analysis (Fry et al., 1978). However, these approaches are limited in their accuracy, and contemporary molecular methods can provide higher resolution of ecological interactions (Zhu et al., 2019).

Molecular methods have been used to characterise species interactions for over 25 years (Asahida et al., 1997; King et al., 2008; Symondson, 2002) and have helped to provide insights into dietary flexibility (Schallhart et al., 2012), niche partitioning (Kartzinel et al., 2015) and the role of biotic interactions in plant invasions (Allen et al., 2021), among other research areas. Various approaches have been employed, ranging from singleplex (Pumariño et al., 2011) and multiplex PCR (Kheirodin et al., 2021; Sint et al., 2016; Staudacher et al., 2011, 2013; Wallinger et al., 2013) through to meta‐barcoding (De la Cadena et al., 2017; García‐Robledo et al., 2013; Wang et al., 2019; Zhu et al., 2019). As the use of molecular methods to infer species interactions has become more commonplace, methodological studies have informed best practice by comparing different approaches. For example, several studies have compared the efficacy of different protocols, including sample collection techniques, DNA extraction methods, storage conditions and PCR recipes (e.g. Oehm et al., 2011; Simonelli et al., 2009; Sint et al., 2011). Other studies have examined ecological factors, such as how plant identity, decomposition level (Wallinger et al., 2013), time post‐feeding (Kamenova et al., 2018; Sint et al., 2018) and meal size (Juen & Traugott, 2005) affect the detection of plant or animal prey DNA. Although the field has progressed remarkably in such a short period, direct comparisons of different sample types are still largely lacking in the literature, especially for herbivorous insects.

Several types of samples are used to study plant–herbivore interactions with molecular methods. A recent review of molecular approaches for dietary analysis of insect herbivores identified the most prevalent type of sample as whole‐body extraction (64% of studies), with the remaining 36% of studies using isolated guts, various body parts (e.g. thorax) and regurgitate (Avanesyan et al., 2021), although faecal samples were not included in the review. Qualitative differences among sample types are important to consider when designing experiments and sampling protocols. For example, regurgitate samples can be collected immediately, whereas obtaining a faecal sample may require housing the organism for an indefinite period. Moreover, using multiple sample types may not be feasible, meaning that the efficacy and biases of different sample types will need to be considered.

Sample type selection is also important for several other reasons. First, DNA may be easier to extract and amplify from some sample types than others. For example, DNA in faecal samples may be more degraded than DNA in gut contents or regurgitate samples because it has been through the entire digestion process. This hypothesis was supported by Sint et al. (2015), who found that prey DNA detection success was significantly lower in faecal than in whole‐body samples of wolf spiders. Similarly, Kamenova et al. (2018) showed that prey DNA from regurgitate and whole‐body samples of carabid beetles was detectable for a longer time post‐feeding than from faecal samples. Overall, both regurgitate and faecal samples appear to provide similar detection rates compared to whole‐body samples (Durbin et al., 2012; Egeter et al., 2015; Unruh et al., 2016), but have the advantages that they are non‐destructive and contain less consumer DNA. However, no studies to date have compared DNA detection success of different sample types from herbivorous insects. Second, some sample types may be biased towards the detection of taxa with certain traits. For example, the DNA of woody plant species or those with tougher leaves may be less degraded after the digestion process, meaning that their detection success in faecal samples could be disproportionately higher than those with more tender leaves. Similarly, exotic plants may be less likely to be fully digested by native herbivores than native plants, due to lower palatability and a lack of coevolutionary history with invertebrate herbivores and their gut microbiome. Third, some insect herbivores exhibit strong sexual dimorphism and differences in foraging rates and host plant preferences between sexes (e.g. White & Watson, 1972), which may also lead to differences in detectability of environmental DNA (eDNA). Thus, it remains unclear whether DNA detectability and host plant identification success differ between sample types and herbivore sexes, whether the use of multiple sample types provides redundant or complementary information, and the traits of plant species are related to their detection frequency in different sample types.

In this study, we compared the use of regurgitate and faecal samples to infer the diet of New Zealand alpine grasshoppers, *Paprides nitidus* Hutton (Orthoptera: Acrididae), in experimental grassland mesocosm communities. Specifically, we tested the following hypotheses: (1) DNA amplification success, RFLP band production, RFLP identification success and the number of identified host plants per sample will be lower for faecal than regurgitate samples; (2) DNA amplification success, RFLP band production, RFLP identification success and the number of identified host plants per sample will be lower for male than the almost three‐fold heavier female grasshoppers; (3) faecal and regurgitate samples will provide similar information about herbivore diet (i.e., host plant identity); and (4) plant species with woody growth form, low specific leaf area and exotic provenance will be more likely to be identified from faecal samples than expected by chance.

## 2 MATERIALS AND METHODS

### 2.1 Experimental design

We manipulated and measured plant–herbivore interactions in 80 mesocosm grassland communities (Allen et al., 2021; Waller et al., 2020). Each mesocosm consisted of a 125 L steel pot, with a bottom layer of 22 L of gravel to aid drainage out of the open bottom, 88 L of pasteurized soil and sand (50:50 mixture) and a top layer of 12 L of soil inoculum (see Waller et al., 2020 for details of soil treatments, which were not considered for this study). Mesocosms were planted with one of 20 unique communities, each consisting of eight plant species (Appendix S1) selected from a pool of 39 plant species that co‐occur in New Zealand grassland communities (19 natives, 20 exotics, Appendix S2). Plants were grown from seed or cuttings collected from New Zealand's South Island (see Waller et al., 2020 for propagation details) and seedlings were randomly positioned in a ring, equally spaced around the centre of the pot during March 2017. Plant communities were replicated four times, with consistent positioning of plant species within each community, and with replicates arranged together to minimize any environmental gradients.

Invertebrate herbivore populations were deliberately established in the mesocosms, which were covered with large mesh cages (0.58 mm Cropsafe Mesh, 15% shade factor, Cosio Industries, Auckland, New Zealand) to keep added herbivores enclosed. One of the herbivore species that was added to the mesocosms was the alpine grasshopper *P. nitidus*. *Paprides nitidus* is an endemic short‐horned grasshopper found in alpine and sub‐alpine habitat in the northern half of the South Island of New Zealand. Based on the Plant‐SyNZ Database (https://plant‐synz.landcareresearch.co.nz/), *P. nitidus* grasshoppers are generalists that have been recorded feeding on 72 host plant species in New Zealand. *Paprides nitidus* grasshoppers exhibit strong sexual dimorphism, with females weighing 2.7 times more than males on average (W. J. Allen, unpublished data) and consuming 2.4 times as much plant material (White & Watson, 1972). Adult grasshoppers were collected from North Canterbury, New Zealand (42°27′ S, 172°49′ E). A male and female pair were first added to the centre of each mesocosm during 8–30 May 2017. Replacement additions occurred after each herbivore survey when either grasshoppers were not observed or were found deceased. Occasionally, this resulted in more than two grasshoppers being sampled from a single mesocosm, because of a false negative on the previous survey (i.e. the grasshopper was present but not observed). In these instances (*n* = 24), we also collected samples from the additional grasshopper, which was then removed from the mesocosm plant community.

### 2.2 Molecular analysis of plant–herbivore interactions

Because the grasshoppers were highly mobile and host plant range could not always be reliably identified from direct observation, we used molecular analysis of DNA from grasshopper faecal and regurgitate samples to determine their host plants. Specifically, we used restriction fragment length polymorphism (RFLP), sometimes known as Amplified rDNA Restriction Analysis (ARDRA) (Gardes et al., 1991). This technique uses restriction enzymes to cut amplified DNA at enzyme‐specific cutting sites, producing different sized DNA fragments that can be used to distinguish among genotypes, species or broader taxonomic groups. By incubating samples overnight with up to three different restriction enzymes, we produced DNA fragment size combinations unique to the eight species in each of the 20 mesocosm communities, which were then visualized on agarose gel (25–766 bp resolution) and cross‐referenced against a database of known samples. RFLP was considered well suited as a technique, given the low diversity of potential host plants (maximum of eight species, all of known identity), and allowed rapid and inexpensive identification of host plants.

### 2.3 Sample collection and storage

**FIGURE 1:**
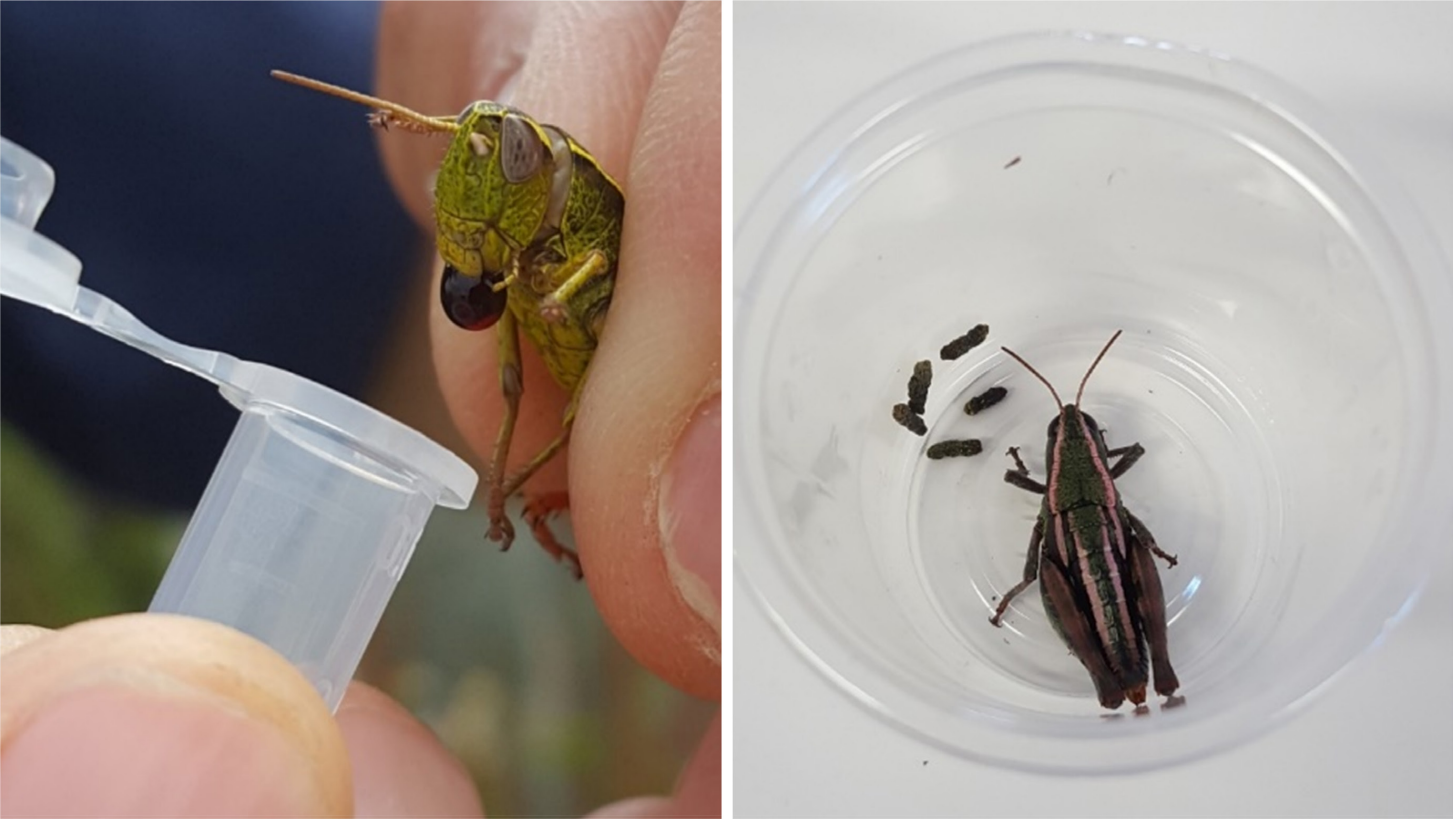
Collection of regurgitate and faecal samples from alpine grasshoppers (*Paprides nitidus*).

Grasshoppers were non‐destructively sampled by collecting regurgitate and faecal samples on four occasions over the one‐year duration of the experiment: June and November in 2017, and January and April in 2018. Up to four individuals were sampled per mesocosm on each sampling occasion (i.e. the number that were added to each mesocosm), depending on how many grasshoppers could be located during a 5‐min search period. Because there were generally only two grasshoppers per mesocosm, and males and females exhibit extreme sexual dimorphism, grasshopper identity and sex were tracked throughout the experiment. Regurgitate was collected by catching and handling grasshoppers, which frequently produced a small bubble of regurgitate as a defensive response to being handled. Samples were collected into 1.7 mL Eppendorf tubes by holding their mouthparts to the lip of the tube (Figure 1). However, not all grasshoppers produced regurgitate and so on some occasions a sample could not be collected. All grasshoppers were then housed in clean rearing cups until they produced faeces (Figure 1), which was collected into Eppendorf tubes using sterile forceps, before the grasshopper was returned to its mesocosm. All samples were stored in a −80°C freezer until DNA extraction. The volume of regurgitate and faeces produced by grasshoppers was highly variable. We did not measure sample volume, although it was observed to be substantially higher for faeces than regurgitate, and for the heavier female than male grasshoppers (W. J. Allen, personal observation).

### 2.4 DNA extraction and polymerase chain reaction (PCR)

DNA was extracted from entire grasshopper faeces and regurgitate samples using Sigma‐Aldrich REDExtract‐N‐Amp kits. Before extraction, regurgitate samples were thawed and centrifuged (30 s at 5600 *g*) to move the regurgitate sample from the lip of the tube to the bottom. Extraction was conducted by first adding 100 μL of extraction solution to each sample. At this point, a sterile pestle was used to crush faecal samples, which were also vortexed and centrifuged (30 s at 5600 *g*). Samples were then heated for 10 min at 95°C. Once cool, 300 μL of dilution solution was added to each sample, which were vortexed and centrifuged (1 min at 5600 *g*). To reduce PCR inhibition, grasshopper faecal and regurgitate samples were further diluted by 1:10 using dilution solution.

The primers used for all PCRs were ITS2F (5′‐ATGCGATACTTGGTGTGAAT‐3′) and ITS3R (5′‐GACGCTTCTCCAGACTACAAT‐3′) (Chen et al., 2010). Each reaction used 11 μL of REDExtract‐N‐Amp PCR mix, 2 μL of each primer (10 μM), 0.5 μL of template DNA and 4.5 μL of PCR grade water, for a total reaction volume of 20 μL. All PCRs were run on Applied Biosystems Veriti PCR machines with a 5‐min ramp up period to 94°C, 40 cycles of 30 s at 94°C, 30 s at 56°C and 45 s at 72°C, followed by an annealing period of 10 min at 72°C, before final cooling to 4°C. Negative and positive controls (*Lolium perenne* DNA) were included in each PCR to test for contamination or PCR failure. Amplicons (400–600 bp) were confirmed with gel electrophoresis on 1% agarose with RedSafe™ dye (Sigma‐Aldrich) in 0.5× TBE buffer for 30 min at 100 V and 500 mA. We loaded 3 μL of PCR product and used 3 μL of Hyperladder 1 kb to estimate amplicon size. Gels were visually examined using a Uvidoc HD2 UV photo machine.

### 2.5 Restriction fragment length polymorphism (RFLP)

We used the restriction enzymes TaqI, HaeIII and MluCI, selected based on virtual digestions using Sanger sequences for each mesocosm plant species and obtained from New England BioLabs (Ipswich, Massachusetts). TaqI reactions used 2 μL of 1× CutSmart® Buffer, 0.5 μL (10 units) of restriction enzyme, 8 μL of PCR product and 9.5 μL of PCR grade water, for a total reaction volume of 20 μL. HaeIII and MluCI reactions used 2 μL of 1× CutSmart® Buffer, 1 μL (10 units) of restriction enzyme, 8 μL of PCR product and 9 μL of PCR grade water, for a total reaction volume of 20 μL. Samples were incubated overnight at 65°C for TaqI and 37°C for HaeIII and MluCI. The resulting fragments were visualized on 2.5% Invitrogen™ UltraPure™ low melting point agarose with RedSafe™ dye in 0.5× TBE buffer for 70 min at 100 V and 500 mA. We loaded 8 μL of digestion product mixed with 2 μL of 6× purple gel loading dye (New England BioLabs) into each lane.

**FIGURE 2:**
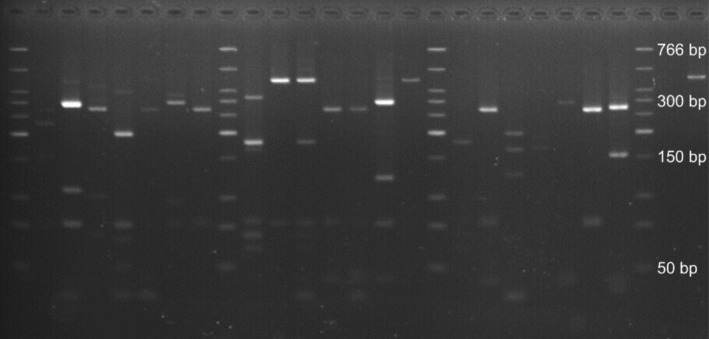
Example RFLP gel showing 25–766 bp DNA ladder (lanes 1, 9, 17 and 25) and banding patterns from digestion with restriction enzyme HaeIII that were used to identify host plant species of the alpine grasshopper *Paprides nitidus*.

Gels were visualized using the Uvidoc HD2 UV photo machine (Figure 2) and band size was estimated by comparison to 5 μL of low molecular weight DNA ladder (25–766 bp, New England BioLabs). Patterns of band sizes were cross‐referenced against our database of virtual digests of Sanger sequences for each plant species to identify the host plant (or plants) in the sample. The database was constructed by searching for restriction enzyme cleavage sites in a representative Sanger sequence for each plant species, and calculating expected fragment lengths. The observed RFLP banding patterns were then confirmed by sequencing of representative samples. Some samples were also sequenced to confirm RFLP identification of samples with faint bands, or to deal with ambiguities in identification based on DNA fragment sizes (GenBank accession numbers OR356011–OR356029). For example, we were unable to reliably distinguish between *Acaena caesiiglauca* and *A. inermis* using RFLP because of their high sequence similarity in the ITS region, and these samples had to be sequenced in communities where they cooccurred. Five samples were identified as containing host plant species that were not intentionally planted (bonfire moss, *Funaria hygrometrica*; parsley‐piert, *Alchemilla arvensis*; and toad rush, *Juncus bufonius*), and were excluded from analyses. These species were likely self‐introduced from the surrounding environment and went unnoticed due to their small stature, with bonfire moss a common greenhouse contaminant and parsley‐piert a widespread weed throughout New Zealand.

### 2.6 Statistical analyses

To compare DNA amplification success, RFLP band production (i.e. a banding pattern produced on the gel) and RFLP identification success (i.e. further sequencing not required to resolve ambiguous or faint banding patterns) between faecal and regurgitate samples, we used a general linear model with amplification success/failure or RFLP band production and identification success/failure as response variables, sample type, grasshopper sex and their interaction as explanatory variables, grasshopper identity and mesocosm as random effects to account for the lack of independence in repeated sampling from the same grasshopper and mesocosm, and a binomial error structure. To compare the number of host plant species identified between faecal and regurgitate samples where plant DNA was successfully amplified, we used a general linear model with number of species identified as the response variable, sample type, grasshopper sex and their interaction as explanatory variables, grasshopper identity and mesocosm as random effects, and a Poisson error structure.

To test whether the overall observed frequencies of identification in faecal samples for plant species differed from expected frequencies, we used a Chi‐squared contingency table test. Finally, to test whether plant species deviances from expected frequency of identification in faecal samples were related to plant species traits, we first focussed the dataset on the 29 plant species that were identified from at least five total samples. We then used simple linear regression with deviance from expected frequency as a response variable, and plant provenance (native, exotic), functional group (herbaceous, woody) and specific leaf area (previously measured by Waller et al., 2020) as explanatory variables. The expected frequency was the overall proportion of positive identifications that came from faecal samples across the experiment (0.66). Deviance from expected frequency was calculated as the proportion of positive identifications for each plant species that came from faecal samples minus the expected frequency. Specific leaf area is a plant trait defined as the ratio of leaf area to dry mass, and that is considered indicative of growth strategy, where high values are associated with low investment in leaf tissue, fast growth rates (Wright et al., 2004) and high palatability (Pérez‐Harguindeguy et al., 2003). All analyses were performed in R 4.1.3 (R Core Team, 2022) using the lme4 (Bates et al., 2019) and emmeans (Lenth et al., 2019) packages.

## 3 RESULTS

**FIGURE 3:**
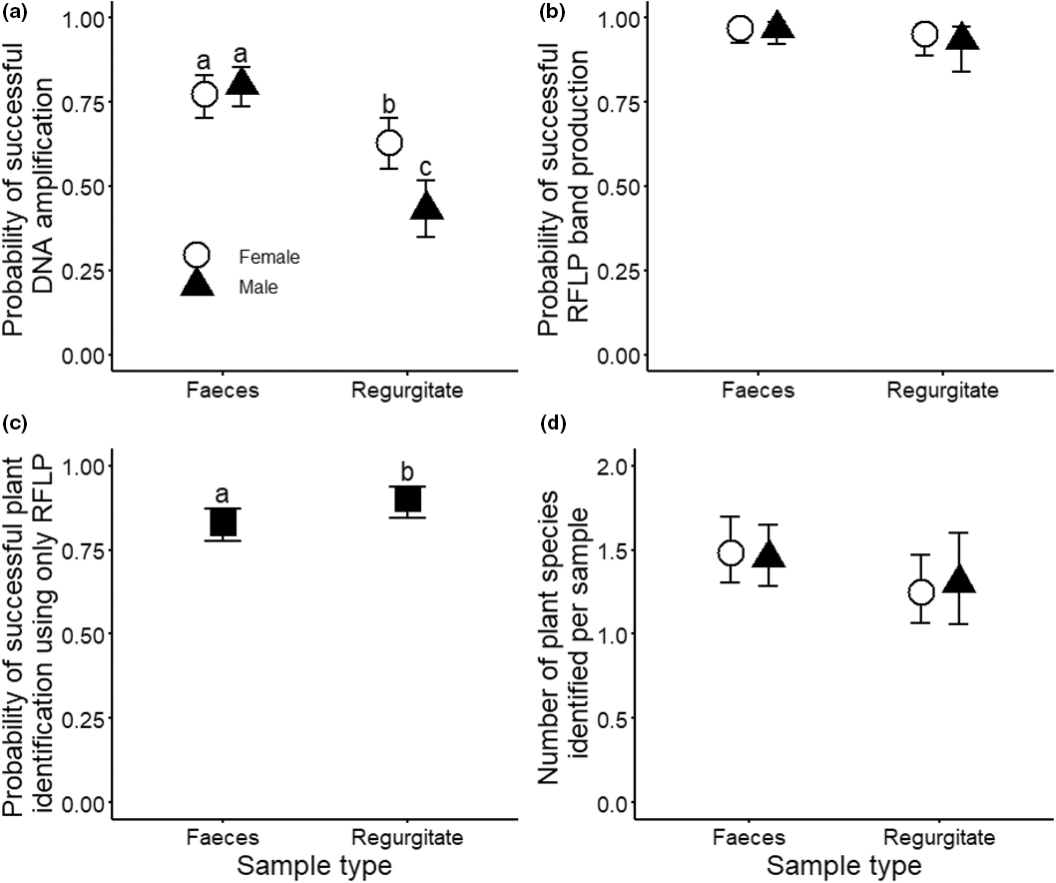
For samples of faeces and regurgitate from female (circles) and male (triangles) alpine grasshoppers (*Paprides nitidus*), the panels present the estimated marginal mean (±95% CI) probability of successful DNA amplification (a), probability of successful restriction fragment length polymorphism (RFLP) band production for samples from which plant DNA was successfully amplified (b), probability of successful host plant identification using only RFLP for samples from which plant DNA was successfully amplified (c) and number of host plant species identified for samples from which at least one species was identified (d). Different lowercase letters indicate significant differences (*p* ≤ .05) between sample types and/or sexes, and a lack of lowercase letters indicates that no significant differences among means were detected using the generalised linear mixed model.

Plant DNA was successfully extracted and amplified from 531 (*n* = 330 faecal; *n* = 201 regurgitate) of the 801 total samples (66%). DNA amplification success depended on the interaction between sample type and grasshopper sex (*Χ* ^2^ = 8.91, *p* = .003). The probability of successful plant DNA amplification was 23% and 86% higher (in relative terms) for faecal than regurgitate samples for female (faecal: 0.773 [0.704, 0.831]; estimated marginal mean [95% CI]; regurgitate: 0.630 [0.552, 0.702]; *p* = .015; Figure 3a) and male (faecal: 0.801 [0.737, 0.853]; regurgitate: 0.431 [0.350, 0.515]; *p* < .001; Figure 3a) grasshoppers, respectively. The probability of successful plant DNA amplification was 46% higher for regurgitate samples from female than male grasshoppers (*p* = .003; Figure 3a), but no sex difference was observed for faecal samples (*p* = 1.000).

RFLP or a combination of RFLP and sequencing identified at least one host plant species from 502 of the 531 samples (95%) from which plant DNA was amplified. Of these 502 samples, 448 (89%) used only the RFLP protocol for identification, and 54 (11%) used a combination of RFLP and sequencing due to ambiguous or faint banding patterns. Success of RFLP in producing bands did not differ significantly between faecal and regurgitate samples (*Χ* ^2^ = 2.60, *p* = .107; Figure 3b), male and female grasshoppers (*Χ* ^2^ = 0.28, *p* = .598; Figure 3b), or depend on their interaction (*Χ* ^2^ = 0.10, *p* = .750; Figure 3b). Success of solely using RFLP for identification was 9% higher for regurgitate (0.901 [0.8454, 0.938]) than faecal (0.829 [0.775, 0.873]) samples (*Χ* ^2^ = 5.06, *p* = .024; Figure 3c), but did not differ significantly between male and female grasshoppers (*Χ* ^2^ = 0.45, *p* = .501) or depend on their interaction (*Χ* ^2^ = 0.02, *p* = .894).

A single host plant species was identified from 335 samples, two host plant species from 133 samples, three host plant species from 31 samples and four host plant species from a single sample. The number of host plant species identified per sample did not differ significantly between faecal and regurgitate samples (*Χ* ^2^ = 3.26, *p* = .071; Figure 3d), male and female grasshoppers (*Χ* ^2^ = 0.001, *p* = .994; Figure 3d), or depend on their interaction (*Χ* ^2^ = 0.15, *p* = .696; Figure 3d).

*Paprides nitidus* were recorded feeding on 38 plant species in the mesocosm experiment, with the only exception being *Chionochloa conspicua*, a grass species that did not survive for long in any of the eight mesocosms that it was planted into. Of the 136 samples for which there were paired regurgitate and faecal samples (i.e. obtained from the same grasshopper during the same sampling period, with successful DNA amplification and RFLP identification of at least one host plant species), only 56 (41%) produced exactly identical host plant identifications, while 100 (74%) were at least partial matches (i.e. matched for at least one of multiple identified host plant species).

**FIGURE 4:**
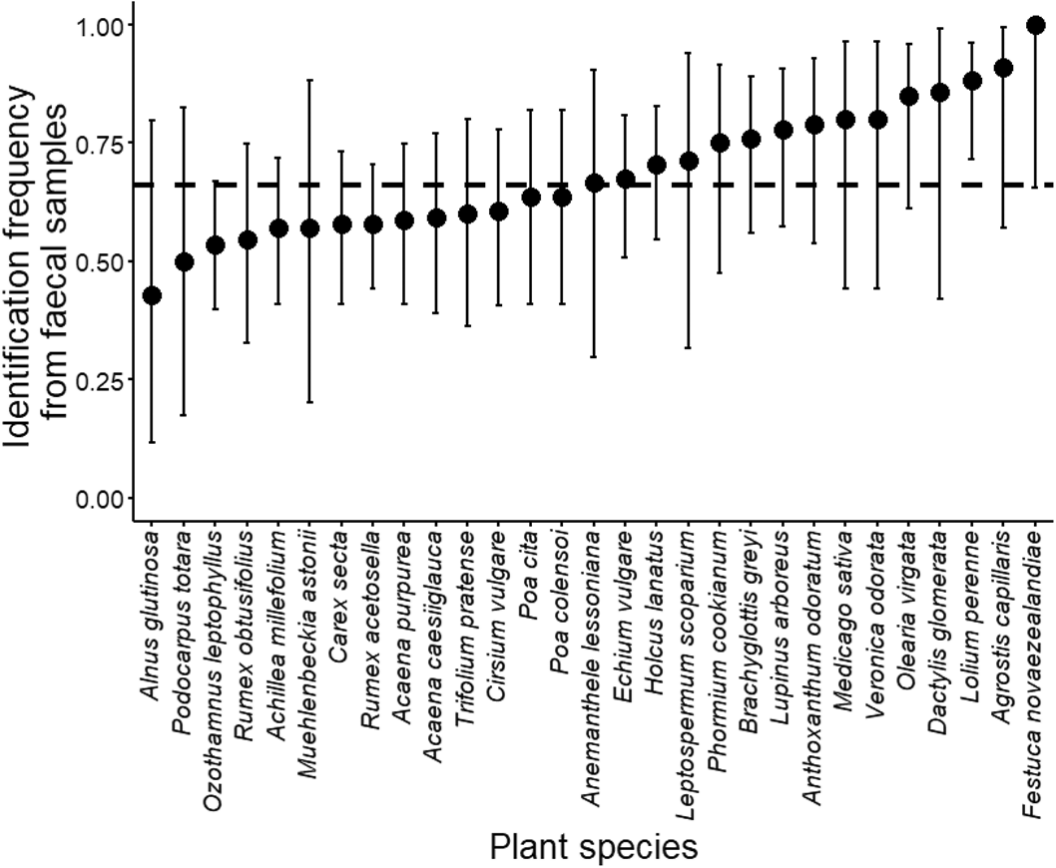
The proportion (±95% CI) of total host plant identifications from faecal samples from alpine grasshoppers (*Paprides nitidus*) for the 29 plant species that were identified from at least five total samples. The horizontal dashed line represents the expected identification from faecal samples based on the overall proportion of faecal samples collected through the experiment. Deviation from expected frequency is the vertical distance between the horizontal line and each data point.

**FIGURE 5:**
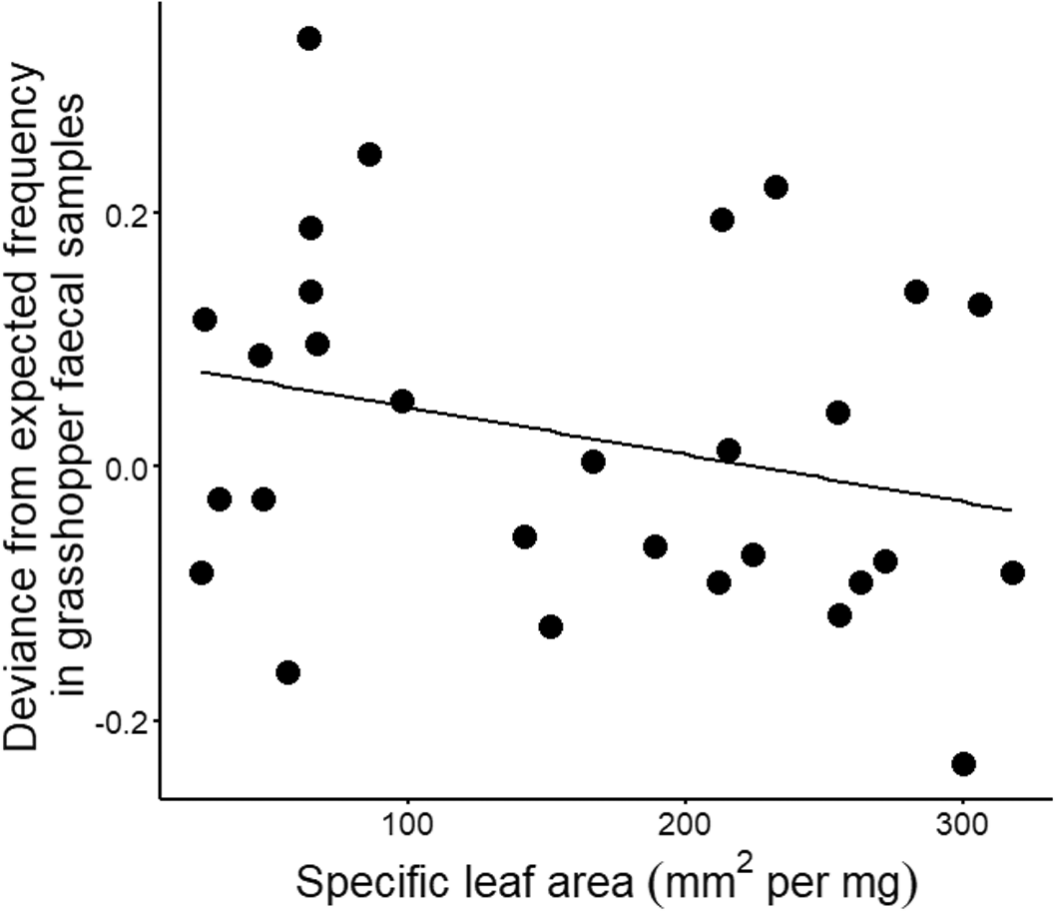
Deviance from expected host plant species identification frequency in alpine grasshopper (*Paprides nitidus*) faecal samples (i.e. values >0 are species represented in faecal samples more than would be expected by chance) as a function of specific leaf area, a proxy for plant growth strategy (i.e. high values represent plant species with lower investment in leaf tissue).

For the 29 plant species that were identified from at least five total samples, plant species demonstrated strong variation in their tendency to be detected from faecal or regurgitate samples than would be expected by chance alone (Figure 4), and a Chi‐squared test supported that observed frequencies differed from expected values overall (*Χ* ^2^ = 41.79, *p* = .045). However, only *Lolium perenne* had a 95% confidence interval that did not overlap with the expected frequency, being identified more often from faecal samples than expected by chance. Moreover, deviance from the expected frequency of occurrence in faecal samples declined with increasing specific leaf area of plant species (*t* = −2.16, *p* = .041; Figure 5). In other words, plants that invested more in leaf tissue biomass were more likely to be identified from faecal samples than expected by chance. Deviance from the expected frequency of occurrence in faecal samples did not differ between native and exotic plants (t = −1.39, *p* = .177) or between woody and herbaceous plants (*t* = −0.80, *p* = .429).

## 4 DISCUSSION

With the burgeoning use of eDNA and molecular analyses to infer species interactions, it is increasingly important to understand how sample collection methods might bias results. Our study helps to address this by presenting the first comparison of plant DNA detectability, host plant species identification frequency and dietary information (i.e. host plant identity) from regurgitate and faecal samples from an insect herbivore. We show that faecal samples yielded higher plant DNA detectability than regurgitate samples for both female and male grasshoppers, while plant DNA detectability from regurgitate samples was higher for female than male grasshoppers. Moreover, successful host plant identification using only RFLP was higher for regurgitate than faecal samples, regardless of grasshopper sex. In contrast, production of RFLP bands and the mean number of host plant species identified per sample did not depend on sample type or grasshopper sex. Importantly, host plant species identification frequency and dietary information differed between sample types taken from the same individual grasshopper, suggesting that faecal and regurgitate samples provide complementary rather than redundant information. Moreover, the host plant species identification frequency from each sample type was related to specific leaf area, indicating that plant traits may influence identification bias from certain sample types. Below we discuss the implications of these findings in the context of best practice for molecular analysis of species interactions.

Despite the host plant tissue having fully passed through the digestion process, DNA amplification success was 23% and 86% higher from faecal than regurgitate samples for female and male grasshoppers, respectively, in contrast to our first hypothesis. One potential explanation for this difference could be the presence of compounds that dilute the sample or inhibit PCR in regurgitate but not faecal samples. Grasshopper regurgitate can contain a mixture of water, partially digested plant tissue, digestive enzymes and salivary secretions such as caeliferins, a type of fatty acid (Alborn et al., 2007; Freeman, 1967, 1968; Lymbery & Bailey, 1980). However, it is unclear which of these components would be both unique to regurgitate and also inhibitory, and we did not test for the presence of inhibitors in the two different sample types. Another potential explanation is that faeces are largely made up of undigested plant material, whereas regurgitate may contain only trace amounts of host plant tissue, depending on when the grasshopper last fed. Previous studies have demonstrated that time post‐feeding can influence the detectability of plant DNA in regurgitate, although Kamenova et al. (2018) also found that prey DNA from regurgitate of carabid beetles was detectable for a longer time post‐feeding than from faecal samples. The authors speculated that this was because regurgitate contained food items that had been more recently consumed, and was therefore less digested resulting in reduced degradation of prey DNA. Alternatively, the volume of faeces tended to be substantially more than that of regurgitate (W. J. Allen, personal observation). This means that the raw amount of DNA in faeces could also be higher, which may help explain why DNA detectability was higher for faecal samples in our study. Female grasshoppers also tended to produce a greater volume of regurgitate than males (W. J. Allen, personal observation), which may help to explain why DNA detectability was higher for regurgitate samples from female than male grasshoppers, the only support for our second hypothesis. Finally, our overall DNA amplification success rate of 66% was fairly low. This could be due to several factors, including the small volume of some samples and the potential presence of PCR inhibitors. Although we were not able to disentangle the mechanisms underlying the observed differences in amplification success, experiments to test the influence of sample volume, PCR inhibitors and other factors on amplification and RFLP success would be a valuable part of future studies that investigate differences among sample types. Future studies may also consider testing how modifications to the protocol affect results, such as the inclusion of duplicate PCR or nucleic acid purification steps.

In contrast to the results for DNA detectability, we found that RFLP identification success was 9% higher for regurgitate than faecal samples, partially supporting our first hypothesis. This result may be due to the presence of additional host plant species in faecal samples in very low concentrations, leading to ambiguous or faint banding patterns that required further sequencing to try and identify host plants, which was not al. This explanation is further supported by the lack of a difference in RFLP success (in terms of producing bands on the gel) between faecal and regurgitate samples. In other words, faecal samples produced RFLP banding patterns at the same rate as regurgitate samples, but these banding patterns were more challenging to interpret without further sequencing.

In contrast to our first hypothesis, the number of host plants identified did not differ between sample types. This finding indicates that once DNA has been successfully amplified, there were no appreciable differences between the two sample types in the number of host species that can be identified using RFLP. Previous studies have suggested that faecal samples can provide a more complete picture of an individual's diet as a single faecal sample may contain the remains of several meals consumed across multiple days (Cheeseman & Gillott, 1987; Deagle et al., 2005). However, this would depend on the speed of digestive metabolism, and faeces could correspond to a single meal for some organisms. Instead, our results indicated that regurgitate and faecal samples provided complementary rather than redundant information about alpine grasshopper diet, contrary to our third hypothesis. A potential explanation for this result could be that the two sample types represent separate feeding events. Alternatively, regurgitate samples may include plants that were only tasted, whereas faecal samples could represent a subset of plants that were consumed in larger quantities. However, 33 and 37 different plant species were identified from regurgitate and faecal samples, respectively, indicating that host plant species richness was high for both sample types. Furthermore, species traits may influence detectability in different sample types. Indeed, some plant species, especially grasses, were more likely to be detected in faecal samples than expected by chance. This detection bias was weakly explained by increased investment in leaf tissue (i.e. low specific leaf area), partially supporting our fourth hypothesis. This result is similar to previous studies that have employed morphological analysis of gut contents, such as McInnis et al. (1983), who showed that grasses were disproportionately represented in comparison to forbs in faecal samples of sheep (*Ovis aries*), likely because their toughness meant that they had lower digestibility. Taken together, these findings indicate that some sample types may be biased towards the detection of species with certain traits.

In this study, we used RFLP to identify host plants that were consumed by *P. nitidus*. Many studies that have characterised plant‐herbivore interactions using eDNA have used downstream techniques such as PCR, multiplex PCR, Sanger sequencing (i.e. barcoding) or meta‐barcoding (Avanesyan et al., 2021). RFLP has previously been used to identify plants from mixed samples (Bobowski et al., 1999; Ridgway et al., 2003), but to the best of our knowledge, this is the first time the technique has been applied to characterise plant‐herbivore interactions. RFLP analysis allows the identification of multiple plant species within single samples, whereas Sanger sequencing is restricted to single‐species samples. The very low cost of RFLP analysis (~$3 NZD per sample) also allows for higher replication at a fixed budget compared to meta‐barcoding. However, RFLP will not be appropriate in all situations. It worked well for this experiment because there were a limited number of options of host plants in each mesocosm community (i.e. a maximum of eight), which allowed them to be distinguished using restriction enzyme digestion. Prior knowledge of potential dietary choices also allowed us to optimise restriction enzyme choice through in silico digestion. The use of RFLP would be less effective in field surveys where there are many possible host plant options, especially when the consumer is highly generalist, or where there are many closely‐related host plant species, which may be more difficult to distinguish using RFLP. For example, we were unable to reliably distinguish between *A. caesiiglauca* and *A. inermis* because of their high sequence similarity. Thus, samples that presented an *Acaena* sp. banding pattern in communities where they cooccurred could only be identified through sequencing. Moreover, the lower detection limit for RFLP is fairly high, meaning that it will not be suitable for small amounts of DNA. For example, the RFLP approach could obscure plant species consumed in very low quantities and which were not the dominant signal in PCR or Sanger sequencing. Therefore, we suggest that RFLP can be a useful and cost‐effective tool for experiments such as feeding trials, choice experiments or when the consumer is a relative specialist in their diet.

Our study adds to the body of literature that informs best practice for molecular analysis of eDNA for characterising the diet of invertebrate herbivores and predators. Our results indicate that faecal samples have higher DNA amplification success than regurgitate samples, and therefore may provide more dietary information per sampling effort. Moreover, most invertebrate herbivores do not produce regurgitate, suggesting that faecal samples may provide a more reliable source of eDNA across a broader diversity of taxa. However, we also show that multiple sample types may be required to fully characterise a species' diet when using molecular analyses of eDNA. Furthermore, the likelihood of detection of host plant species in different sample types may depend on plant traits (e.g. specific leaf area). Therefore, we recommend that future studies should use faecal samples to maximise the dietary information, and that multiple sample types should be used if the objective is to fully characterise a species' diet. Finally, future research is urgently needed to further understand the potential for detection bias in different types of eDNA samples, as well as the traits that underly these biases.

## AUTHOR CONTRIBUTIONS

**Warwick J. Allen:** Conceptualization (equal); data curation (equal); formal analysis (lead); investigation (equal); methodology (equal); writing – original draft (lead); writing – review and editing (equal). **Lauren P. Waller:** Conceptualization (equal); data curation (equal); investigation (equal); methodology (equal); writing – review and editing (equal). **Barbara I.P. Barratt:** Conceptualization (equal); funding acquisition (supporting); methodology (equal); supervision (equal); writing – review and editing (equal). **Ian A. Dickie:** Conceptualization (equal); funding acquisition (lead); methodology (equal); project administration (lead); supervision (equal); writing – review and editing (equal).

## CONFLICT OF INTEREST STATEMENT

The authors declare no competing interests.

## Supporting information


Appendix S1.
Appendix S2.

## Data Availability

Data are available for download from Dryad: https://doi.org/10.5061/dryad.1rn8pk10p. Sequences are deposited in GenBank (accession numbers OR356011–OR356029). Code available on request from the corresponding author.
