# Supplementary material for: Puke or poop? Comparison of regurgitate and faecal samples to infer alpine grasshopper (Paprides nitidus Hutton) diet in experimental plant communities
Source: Ecol Evol. 2023 Aug 29;13(8):e10444. doi: 10.1002/ece3.10444 (PMC10463123; doi:10.1002/ece3.10444)
Supplement: Supplementary file 1 — Appendix S1. Appendix S2. [file ECE3-13-e10444-s001.docx]

**Appendix S1.** Plant species composition of each mesocosm community.

| Community | Plant species | Community | Plant species | Community | | Plant species |  |
| --- | --- | --- | --- | --- | --- | --- | --- |
| 1 | *Agrostis capillaris* | 2 | *Achillea millefolium* | 3 | | *Acaena inermis* |  |
| 1 | *Anthoxanthum odoratum* | 2 | *Cirsium vulgare* | 3 | | *Anthoxanthum odoratum* |  |
| 1 | *Holcus lanatus* | 2 | *Dactylis glomerata* | 3 | | *Brachyglottis greyi* |  |
| 1 | *Hypericum perforatum* | 2 | *Echium vulgare* | 3 | | *Chionochloa conspicua* |  |
| 1 | *Lolium perenne* | 2 | *Festuca novae-zelandiae* | 3 | | *Echium vulgare* |  |
| 1 | *Rumex obtusifolius* | 2 | *Medicago sativa* | 3 | | *Poa colensoi* |  |
| 1 | *Trifolium pratense* | 2 | *Ozothamnus leptophyllus* | 3 | | *Rumex acetosella* |  |
| 1 | *Trifolium repens* | 2 | *Rumex acetosella* | 3 | | *Rumex obtusifolius* |  |
| 4 | *Acaena caesiiglauca* | 5 | *Acaena caesiiglauca* | 6 | | *Achillea millefolium* |  |
| 4 | *Anemanthele lessoniana* | 5 | *Acaena inermis* | 6 | | *Cirsium vulgare* |  |
| 4 | *Carex secta* | 5 | *Anemanthele lessoniana* | 6 | | *Holcus lanatus* |  |
| 4 | *Festuca novae-zelandiae* | 5 | *Brachyglottis greyi* | 6 | | *Lolium perenne* |  |
| 4 | *Hypericum perforatum* | 5 | *Festuca novae-zelandiae* | 6 | | *Lupinus arboreus* |  |
| 4 | *Medicago sativa* | 5 | *Ozothamnus leptophyllus* | 6 | | *Pinus contorta* |  |
| 4 | *Phormium cookianum* | 5 | *Phormium cookianum* | 6 | | *Trifolium pratense* |  |
| 4 | *Poa cita* | 5 | *Poa colensoi* | 6 | | *Trifolium repens* |  |
| 7 | *Achillea millefolium* | 8 | *Acacia dealbata* | 9 | | *Anemanthele lessoniana* |  |
| 7 | *Agrostis capillaris* | 8 | *Acaena caesiiglauca* | 9 | | *Anthoxanthum odoratum* |  |
| 7 | *Carex secta* | 8 | *Acaena inermis* | 9 | | *Brachyglottis greyi* |  |
| 7 | *Dactylis glomerata* | 8 | *Alnus glutinosa* | 9 | | *Carex secta* |  |
| 7 | *Hypericum perforatum* | 8 | *Cirsium vulgare* | 9 | | *Coprosma robusta* |  |
| 7 | *Ozothamnus leptophyllus* | 8 | *Phormium cookianum* | 9 | | *Festuca novae-zelandiae* |  |
| 7 | *Pinus radiata* | 8 | *Poa cita* | 9 | | *Muehlenbeckia astonii* |  |
| 7 | *Ulex europaeus* | 8 | *Trifolium pratense* | 9 | | *Rumex acetosella* |  |
| 10 | *Acaena caesiiglauca* | 11 | *Acacia dealbata* | 12 | | *Alnus glutinosa* |  |
| 10 | *Carex secta* | 11 | *Agrostis capillaris* | 12 | | *Anemanthele lessoniana* |  |
| 10 | *Festuca novae-zelandiae* | 11 | *Dactylis glomerata* | 12 | | *Brachyglottis greyi* |  |
| 10 | *Leptospermum scoparium* | 11 | *Holcus lanatus* | 12 | | *Echium vulgare* |  |
| 10 | *Olearia virgata* | 11 | *Lolium perenne* | 12 | | *Lupinus arboreus* |  |
| 10 | *Ozothamnus leptophyllus* | 11 | *Pinus radiata* | 12 | | *Medicago sativa* |  |
| 10 | *Phormium cookianum* | 11 | *Trifolium repens* | 12 | | *Pinus contorta* |  |
| 10 | *Poa cita* | 11 | *Ulex europaeus* | 12 | | *Rumex obtusifolius* |  |
| 13 | *Acacia dealbata* | 14 | *Acaena inermis* | 15 | | *Acaena inermis* |  |
| 13 | *Achillea millefolium* | 14 | *Anemanthele lessoniana* | 15 | | *Carex secta* |  |
| 13 | *Carex secta* | 14 | *Echium vulgare* | 15 | | *Leptospermum scoparium* |  |
| 13 | *Hypericum perforatum* | 14 | *Holcus lanatus* | 15 | | *Ozothamnus leptophyllus* |  |
| 13 | *Leptospermum scoparium* | 14 | *Muehlenbeckia complexa* | 15 | | *Phormium cookianum* |  |
| 13 | *Poa colensoi* | 14 | *Poa cita* | 15 | | *Poa cita* |  |
| 13 | *Rumex obtusifolius* | 14 | *Podocarpus totara* | 15 | | *Sophora microphylla* |  |
| 13 | *Hebe odora* | 14 | *Sophora microphylla* | 15 | | *Hebe odora* |  |
| 16 | *Agrostis capillaris* | 17 | *Anthoxanthum odoratum* | 18 | | *Alnus glutinosa* |  |
| 16 | *Alnus glutinosa* | 17 | *Coprosma robusta* | 18 | | *Brachyglottis greyi* |  |
| 16 | *Lupinus arboreus* | 17 | *Lolium perenne* | 18 | | *Cirsium vulgare* |  |
| 16 | *Pinus contorta* | 17 | *Lupinus arboreus* | 18 | | *Lupinus arboreus* |  |
| 16 | *Pinus radiata* | 17 | *Ozothamnus leptophyllus* | 18 | | *Muehlenbeckia complexa* |  |
| 16 | *Rumex acetosella* | 17 | *Pinus contorta* | 18 | | *Olearia virgata* |  |
| 16 | *Trifolium pratense* | 17 | *Pinus radiata* | 18 | | *Phormium cookianum* |  |
| 16 | *Ulex europaeus* | 17 | *Ulex europaeus* | 18 | | *Ulex europaeus* |  |
| 19 | *Acaena caesiiglauca* | 20 | *Carex secta* |  | |  |  |
| 19 | *Muehlenbeckia astonii* | 20 | *Muehlenbeckia astonii* |  | |  |  |
| 19 | *Muehlenbeckia complexa* | 20 | *Olearia virgate* |  | |  |  |
| 19 | *Phormium cookianum* | 20 | *Ozothamnus leptophyllus* |  |  | | |
| 19 | *Pinus contorta* | 20 | *Phormium cookianum* |  |  | | |
| 19 | *Pinus radiata* | 20 | *Podocarpus totara* |  |  | | |
| 19 | *Poa colensoi* | 20 | *Sophora microphylla* |  |  | | |
| 19 | *Podocarpus totara* | 20 | *Hebe odora* |  |  | | |

**Appendix S2.** All plant species used in the experiment. Information shown includes plant species’ provenance, functional group, and specific leaf area.

| Plant name | Family | Provenance | Functional group | Specific leaf area (mm² per mg) |
| --- | --- | --- | --- | --- |
| *Acacia dealbata* | Fabaceae | Exotic | Woody | 144.27 |
| *Acaena caesiiglauca* | Rosaceae | Native | Herbaceous | 224.23 |
| *Acaena inermis* | Rosaceae | Native | Herbaceous | 272.24 |
| *Achillea millefolium* | Asteraceae | Exotic | Herbaceous | 263.38 |
| *Agrostis capillaris* | Poaceae | Exotic | Herbaceous | 85.94 |
| *Alnus glutinosa* | Betulaceae | Exotic | Woody | 300.18 |
| *Anemanthele lessoniana* | Poaceae | Native | Herbaceous | 166.85 |
| *Anthoxanthum odoratum* | Poaceae | Exotic | Herbaceous | 306.28 |
| *Brachyglottis greyi* | Asteraceae | Native | Herbaceous | 67.14 |
| *Carex secta* | Cyperaceae | Native | Herbaceous | 25.40 |
| *Chionochloa conspicua* | Poaceae | Native | Herbaceous | 73.58 |
| *Cirsium vulgare* | Asteraceae | Exotic | Herbaceous | 141.72 |
| *Coprosma robusta* | Rubiaceae | Native | Woody | 151.77 |
| *Dactylis glomerata* | Poaceae | Exotic | Herbaceous | 213.02 |
| *Echium vulgare* | Boraginaceae | Exotic | Herbaceous | 215.72 |
| *Festuca novae-zelandiae* | Poaceae | Native | Herbaceous | 64.47 |
| *Hebe odora* | Plantaginaceae | Native | Woody | 65.02 |
| *Holcus lanatus* | Poaceae | Exotic | Herbaceous | 255.14 |
| *Hypericum perforatum* | Hypericaceae | Exotic | Herbaceous | 349.52 |
| *Leptospermum scoparium* | Myrtaceae | Native | Woody | 97.64 |
| *Lolium perenne* | Poaceae | Exotic | Herbaceous | 232.54 |
| *Lupinus arboreus* | Fabaceae | Exotic | Woody | 26.62 |
| *Medicago sativa* | Fabaceae | Exotic | Herbaceous | 283.35 |
| *Muehlenbeckia astonii* | Polygonaceae | Native | Woody | 212.16 |
| *Muehlenbeckia complexa* | Polygonaceae | Native | Herbaceous | 122.16 |
| *Olearia virgata* | Asteraceae | Native | Woody | 64.78 |
| *Ozothamnus leptophyllus* | Asteraceae | Native | Herbaceous | 151.70 |
| *Phormium cookianum* | Asphodelaceae | Native | Herbaceous | 46.86 |
| *Pinus contorta* | Pinaceae | Exotic | Woody | 73.94 |
| *Pinus radiata* | Pinaceae | Exotic | Woody | 42.27 |
| *Poa cita* | Poaceae | Native | Herbaceous | 32.09 |
| *Poa colensoi* | Poaceae | Native | Herbaceous | 47.88 |
| *Podocarpus totara* | Podocarpaceae | Native | Woody | 56.82 |
| *Rumex acetosella* | Polygonaceae | Exotic | Herbaceous | 318.04 |
| *Rumex obtusifolius* | Polygonaceae | Exotic | Herbaceous | 255.68 |
| *Sophora microphylla* | Fabaceae | Native | Woody | 123.65 |
| *Trifolium pratense* | Fabaceae | Exotic | Herbaceous | 189.06 |
| *Trifolium repens* | Fabaceae | Exotic | Herbaceous | 204.57 |
| *Ulex europaeus* | Fabaceae | Exotic | Woody | 23.24 |
